# Recycling in Babel: The Impact of Foreign Languages in Rule Learning

**DOI:** 10.3390/ijerph17113784

**Published:** 2020-05-27

**Authors:** Eneko Antón, Natalia B. Soleto, Jon Andoni Duñabeitia

**Affiliations:** 1 Humanitate eta Hezkuntza Zientzien fakultatea, Mondragon Unibertsitatea, 20540 Eskoriatza, Spain; eanton@mondragon.edu; 2 Centro de Ciencia Cognitiva (C3), Universidad Nebrija, 28015 Madrid, Spain; n.soleto24@gmail.com; 3 Department of Languages and Culture, The Arctic University of Norway, 9019 Tromsø, Norway

**Keywords:** foreign language, recycling, rule learning, decision making, bilingualism

## Abstract

Environmental decisions and prosocial behaviors have been shown to be emotionally mediated, and language is at the core of emotions. The language context can alter the way decisions are made, and using a foreign language tends to favor an analytic approach to the decision and reduce its emotional resonance. In the present work, we explored whether or not the strategic use of a native vs. a non-native language could alter the learning of rules that are at the basis of our environmental behavior. To test this, elementary school students carried out a series of tasks that required recycling the employed materials at the end of the session. Children had to put each kind of material used in the corresponding container following basic association rules, resembling the process that they would do at home when recycling. Some students received the whole set of instructions and rules in their native language, while others received them in their foreign language. When the recycling behaviors were compared, results showed that participants who were instructed in their non-native language followed the rules better than their natively instructed peers. These results are discussed in the light of different perspectives, and future directions in the strategic use of language contexts are considered.

## 1. Introduction

We make decisions on a daily basis in many different areas—political decisions, personal decisions, financial decisions, romantic decisions, career decisions. They can range from trivial (e.g., “what clothes should I wear today?”, “should I have coffee or tea for breakfast?”) to those crucial to our lives and our future (“should I accept that job?”, “should I move to that country?”, “should I invest this money?”). Often, the relevance of the decision is not defined by the question per se, but rather by its context. For example, the question “what clothes should I wear today?” would possibly be of little importance when going for the groceries, but of capital relevance when getting ready for a job interview or an important occasion. Also, the weather may also play a critical role in making this decision.

Thus, simply based on time/effort-efficiency ratios, one might consider it logical that the less important decisions are made following more intuitive and heuristic pathways. In more relevant and important situations, however, we would generally take a more rational, analytic, and rule-governed approach. Indeed, these two approaches to decision making have been extensively studied in the literature [1,2,3]. However, this sharp division in deciding styles is not that crystal clear, as several factors that in principle might seem irrelevant for the question can affect decision making (see, for example, [4]), like the time of the day in which the decision is made, the familiarity with the situation, or its difficulty. Hence, these context factors, as well as individual variable characteristics can have an impact on the outcome of our decisions and on how we approach said decisions, many of which are intuitively considered irrelevant [4]. Surprisingly, the relevance of some of those factors might not be so obvious, which is the case with language. Language is inherent in every decision; we all are constantly embedded in a specific language context. Language is the tool we use to understand the situation, to evaluate the available options in decision-making, to hypothesize about the possible outcomes, and to communicate our decisions. Hence, language is instrumental in every decision we make.

At the same time, hundreds of millions of people in the globalized world of today use more than one language to communicate. We often travel, buy things, and consume media in languages other than our mother tongue. According to the 23rd edition of *Ethnologue* [5], English is the most commonly spoken non-native second language (hereafter L2). Its use has been increasingly growing since the mid-nineteenth century [6], and it has the status of lingua franca, being a language used for global interethnic communication [7]. Worldwide organizations conduct meetings in English and important political decisions are being taken in this language, despite being a non-native language to a big part of its speakers. Hence, understanding how the use of a non-native language could shape decision-making processes, preferences, and choices is crucial.

Recent research has shown that the decisions made in a foreign language tend to be different from those made in a native tongue. This is known as the ‘foreign language effect’ (or FLE for short; see [8,9,10,11]). As previously explained, decisions are often made following principles that vary from intuitive to systematic. In their seminal study, Keysar, Hayakawa, and An [11] argued that the use of a foreign language during decision-making could modulate people’s preference for those principles in two possible ways. On the one hand, second languages are usually harder to use, and therefore, they could increase the cognitive load, involving more cognitive resources. Given that most of our cognitive resources would be allocated to dealing with the foreign language, and consequently, fewer resources would be free for the decision-making processes, choices would be made based on more intuitive processes. According to this approach, decisions made in a foreign language should be more biased, being largely influenced by heuristics and affection. On the other hand, there are also good reasons to believe that the opposite could be true, as foreign languages are less emotionally grounded than native ones [12]. Subjective ratings and even electrodermal responses have shown that participants react less emotionally to taboo words, expressions of love, rebukes, and other emotionally charged stimuli, when these are presented in a foreign language [13,14,15,16,17]. According to this, decisions made in a foreign language should reduce the emotional resonance of the situation and allow people to rely more on systematic mechanisms associated with analytic approaches. To adjudicate between the two alternatives, Keysar, Hayakawa, and An [11] carried out a series of experiments that suggested that people relied more on systematic mechanisms when the decisions were presented in a foreign language context. They concluded that using a foreign language when making a decision could increase the psychological distance and promote deliberation, especially due to the reduction in the emotional implication associated with foreign language use. In recent years, this effect has been recurrently replicated recurrently in the literature [8,18,19].

However, several questions regarding both the origin and the stability of this effect are still subject to debate. For example, Hadjichristidis and colleagues [20] decided to revisit the debate between the two possible outcomes of an FLE proposed by Keysar and colleagues [11], as new data defended both the brain-drain model [21] and views based on improved decision-making [11,22]. The brain-drain model [21] posits that foreign language use during decision making depletes the controlled and conscious cognitive resources normally required to monitor and correct automatic responses, resulting in biases during decision making. In contrast, views based on improved or enhanced decision-making suggest that heuristically generated biases are reduced [11,22]. Hadjichristidis and colleagues [20], after analyzing the available data, defended that both outcomes are possible and that they depend both on the type of task involved and the cognitive process for which the foreign language increases the cognitive load. This way, recent research has focused on the extent and limits of the FLEs, exploring the concrete scenarios in which they can arise, and results are not fully compelling. While some findings indicate that a foreign language context induces less judgment of risk and more positive overall effect when evaluating specific hazards [23], indicating that some emotional valence is required for the effect, other findings report an FLE in situations that are not emotionally charged, such as the judgments of harmful and harmless social norm violations [24], as well as in situations where the FLE was not mediated by an attenuation of emotions [25]. In this line, Vives, Aparici, and Costa [26] considered that there were two possible sources of a decision-making improvement via the FLE. The first source is the reduced emotionality account [11,27], according to which the foreign language reduces the emotional reactivity to the situation. Consequently, the FLE should only be captured in emotionally charged situations, and it should favor an analytic approach. The second source is the cognitive enhancement hypothesis, according to which the FLE directly increases analytic processing [8,11]. Their research suggested that the FLE only emerges in situations where emotions play a causal role, supporting the first approach.

While many researchers have put the effort on trying to disentangle the origins of the FLE, and even though this is still a matter of research and discussion, the question that is capturing the attention of many scholars lately is whether or not one could use languages in a strategic manner to modify behavior [28,29]. If the FLE indeed promotes deliberation and analytic processing, and if this is produced by the reduced emotionality (which, at the moment, seems to be most widely accepted explanation), one could potentially use a foreign language to increase rational and analytic (and less emotional) decision-making processes [29]. One of the contexts in which the strategic use of a foreign language could mitigate intuitive responses in favor of prosocial actions is environmental behavior, where emotions have been shown to have a mediating role (see [30,31]). Pro-environmental behaviors such as recycling are at the core of the strategic decisions made by governments around the world today. Thus, it is worth considering if the strategic use of foreign languages could lead to different degrees of involvement of citizens in pro-environmental actions. Despite the efforts and political campaigns to encourage it, we often hear people defend groundless opinions against it, which could frequently be emotionally biased and/or irrational (e.g., “it´s too much effort,” “it´s not worth it,” “I don´t see any benefit”). Indeed, a survey conducted by Ipsos Public Affairs in 2001 in the U.S. lists “lack of convenience,” “being too time-consuming,” and “they just forget” as the top three reasons why American citizens decided not to recycle [32]. One might wonder if setting a foreign language context for such decisions would boost rational thinking (e.g., a way of thinking based on objective benefits for society, avoiding personal biased opinions) and change the behavior. In the current study, we specifically aimed at exploring whether the compliance of a series of rules related to recycling could be modulated by the language context (native vs. foreign) in which they were learned, as a strategic attempt to shape pro-environmental behavior at school.

Environmental awareness has become a top priority for nations all around the world, as we are experiencing a severe climate change and agreements to stop it or slow it down are part of most governments’ agendas. Recycling is a key factor in sustainable development, and policies focused on encouraging it have become very relevant. Many policies aimed at guaranteeing sustainable development have emphasized the role of education in raising awareness, a sine qua non feature for later development of environment-protective behaviors [33,34]. Thus, the role of education in promoting values, attitudes, skills, and behaviors coherent with a sustainable development perspective is undeniable ([35]; see also [36,37,38]). Particularly, schools play a crucial role in promoting and developing children’s knowledge and attitudes towards sustainability [39,40]. However, the number of studies exploring environmental awareness and behavior of the target population of these policies, children, is not as extensive as one might think. So far, mostly descriptive and exploratory studies have focused on environmental knowledge and behavior [41,42], and the potential impact of different policies and different social factors [43]. Here we adopted a more instrumental perspective and aimed at exploring the role of language of instruction in environmentally friendly behaviors. We investigated if the strategic use of a foreign language during recycling rule instructions could make students follow these rules better. If the effect of a foreign language context during rule learning indeed follows a brain-drain model [21], the approach to recycling should be more biased, and therefore, the rule would be most likely ignored or less followed. If, on the contrary, it enhances decision making [11,22], the rule should be better followed after learning it in a foreign language. This second outcome seems plausible, as it has been repeatedly shown that prosocial behaviors are often emotionally mediated [32], and the emotional distancing and rational thinking potentially induced by a foreign language context could contribute to a better rule learning [26]. Here we focus on the impact of language context in rule learning, given that environmental and prosocial behaviors usually start with the learning of a rule (e.g., recycling), which must be well internalized and learned before following it.

In a nutshell, in the current experiment, elementary school children engaged in a new task that involved a series of rules to recycle the different used materials (namely, putting each kind of material used in its corresponding container, akin to the recycling process that children could do at home). The children were exposed to the instructions in their native language (Spanish), or in their foreign language (English). We explore whether material disposal rules learned in a foreign language context are better complied with.

## 2. Materials and Methods

### 2.1. Participants

Due to the center’s availability and convenience for running the experiment at the moment of the study, ninety students (50 females) from the fifth grade of elementary school at Colegio Mirasur (Madrid, Spain) took part in the experiment. They were either 10 or 11 years old, and they all were native speakers of Spanish and had acquired English as a second language at the mean age of 6. These students were randomly divided into four groups when they entered school (A, B, C, and D groups). Their English language grades were taken as a proxy for their English proficiency, and an ANOVA showed that there were no significant differences between the groups in their knowledge of English (*p* > 0.33). Each group was randomly assigned to a language of instruction condition, resulting in groups A and C (*n* = 47) being assigned to the Spanish (native language) condition, and groups B and D (*n* = 43) being assigned to the English (foreign language) condition. All the participants’ parents or legal guardians gave informed consent before the first experimental session, and they were appropriately informed about the basic procedure of the experiment, according to the ethical commitments established by the Ethics Committee of the Universidad Nebrija that approved the experiment (approval code JADL021020192).

### 2.2. Materials

For the purposes of the experiment, blue, red, yellow, green, white, and black wooden blocks were used as the critical materials to be manipulated, and three containers of different colors (pink, purple, and brown) were used as their recycling bins. Each participant from the Spanish groups received five red blocks, five green blocks, and five white blocks at the beginning of the session. Similarly, each participant from the English groups received five blue blocks, five black blocks, and five yellow blocks. The accuracy in the allocation of the blocks in the recycling bins following the color associations given in the instructions was taken as the dependent variable.

### 2.3. Procedure

The experiment consisted of two experimental sessions that were carried out two non-consecutive days of the same week (e.g., Monday and Wednesday). Both sessions were carried out in school dependencies at school hours.

Day one: On Monday, participants were presented with video-recorded instructions explaining the task they had to perform. The instructions were given in the language corresponding to the language condition assigned to each of the groups, and they concerned the corresponding block colors. This way, groups A and C received a series of instructions in Spanish mentioning red, green, and white colors, and groups B and D received them in English with mentions to the blue, black, and yellow colors. The same Spanish-English bilingual experimenter recorded both video clips. The instructions referred to one distracting task (the building of a tower) and the critical test task that students had to complete (the block-container association task). The instructions were as follows, with the only between-language variations being the language of instruction and the block colors:

“We are going to play a game where you have to build up a tower with all the blocks. The tallest tower gets a prize. You will have five minutes. Once you have finished, you must clean up and put the blocks in the right container. XXXX blocks go into the purple bin, YYYY blocks go in the brown container and ZZZZ pieces in the pink one. Let’s start the game!”

Importantly, XXXX, YYYY, and ZZZZ varied as a function of the language manipulation, mentioning the three different corresponding colors of the blocks. The tower building was a distraction task to get participants’ attention and engagement. Once they completed it, they had to place the blocks in the containers outside the classroom, where the experimenter took notes on their accuracy. The students left the classroom one by one in a pre-determined order to complete the block-container association task (namely, the recycling task), while the teachers made sure that participants who remained in the classroom did not exchange information regarding the block-container association rules. One experimenter was present during the block disposal, but she did not communicate with the students. The containers were closed so that participants could not see what was inside of them, and leaving part of the materials out of the containers was not allowed.

Day 2: On Wednesday, students repeated the whole procedure without receiving any instructions. They built the tower again with the same blocks used on Monday, and after the distraction task, they went outside the classroom to place the blocks in the appropriate containers. The experimenters noted down the accuracy for each participant.

## 3. Results

Accuracy was collected for each participant on each of the two days, and results were averaged for each language of instruction. Accuracy measures were obtained on each test day by considering if each of the 15 color blocks that a participant used during the tower building task was placed in the correct corresponding recycling bin. Hence, each participant could reach a maximum of 15 correct responses (5 for each color), and this would correspond to 100% of accuracy for that day. Participants in the English group reached a mean accuracy rate of 82.2% on Day 1 (standard deviation (SD) = 33.6) and 72.1% on Day 2 (SD = 37.7). Participants in the Spanish group obtained a mean accuracy rate of 54.6% on Day 1 (SD = 41.4) and 56.7% on Day 2 (SD = 39.3).

**Figure 1:**
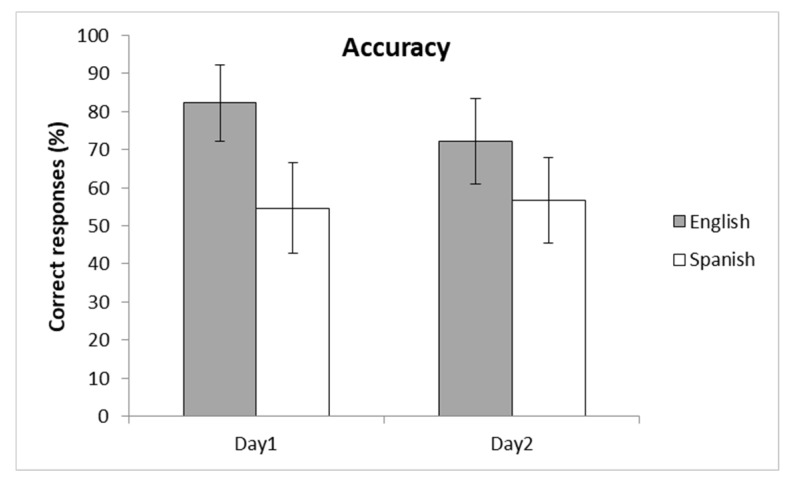
Mean accuracy (percentage of hits) in the block-container association task divided by the language of instruction. Error bars indicate 95% confidence intervals.

The results were analyzed following a 2 × 2 ANOVA, with language (English/Spanish) and day (Day1/Day2) as factors. The results (see Figure 1 and Supplementary Materials) showed that the language factor was significant (F(1, 88) = 9.85, *p* < 0.01), demonstrating that participants who learned the rules in English performed better on both days (mean accuracy of 77.15%) than those who learned them in Spanish (mean accuracy of 55.65%). The Day factor was not significant (F(1, 88) = 0.865, *p* > 0.36), suggesting that participants interiorized the block-container association well and that there was no accuracy drop due to the delayed testing. The interaction between the two factors was not significant (F(1, 88) = 2.04, *p* > 0.16).

In order to explore the absence of interaction in more detail, a Bayesian null hypothesis testing comparison [44,45] was performed, comparing the differences in accuracy between Day 1 and Day 2 for each language group. The null hypothesis (namely, that the differences in accuracy between days were constant across the groups of the language of instruction), and the alternative hypothesis (namely, that the between-day variations would be different for each group) were contrasted using a Bayesian approach. Comparing those two hypotheses, a Bayes Factor BF_10_ of 0.54 was obtained. The Bayes Factor is generally considered to be a quantification of the relative predictive performance of two rival hypotheses [46], and our results clearly indicated that there was no evidence in support of the alternative hypothesis whatsoever, and thus, the data were more likely under the assumption that the null hypothesis was true.

## 4. Discussion

Recycling and other pro-environmental behaviors are key to promoting sustainability. However, despite the efforts of public institutions, recycling is not fully widespread. Thus, it is worth exploring whether the strategic use of certain factors such as language could enhance the learning of rules that are at the basis of different prosocial behaviors, such as recycling, making the citizens more involved in the actions related to environmental awareness. In this article, we aimed at exploring the foreign language effects in recycling-related rule learning. Akin to the rules of the recycling process that every individual can carry out at home, elementary school students received a set of rules on how to dispose of a specific set of materials.

It has been previously shown that environmental behavior is emotionally mediated [31]. For example, anticipating a potentially positive future emotional state (e.g., after-recycling pride) before making the environmental decision results in higher pro-environmental behavioral intentions than anticipating a potentially negative future emotional state (e.g., guilt) from inaction [30]. If emotions play such an important role in prosocial decisions and if the use of a foreign vs. native language can modulate the emotional impact of a given situation [11,26,27], the question is whether or not the strategic use of language can change an individual’s approach to prosocial decision-making. In this line, the seminal study by Geipel et al. [29] showed that consumers’ acceptance of innovative products that help sustainability (such as recycled water, or products that could create disgust, such as insect-based food) improved if they were described in a foreign language.

In our experiment, we followed a similar approach, but we tried to observe the potential impact that language of instruction could have on recycling rule learning. We observed that participants who were instructed in their non-native language followed the rules better than those who were instructed in their native language. In other words, participants who learned the recycling rules in a non-native language performed better than their peers who learned in their native tongue. These results might look surprising, considering that all the students were native speakers of Spanish and that their English knowledge, although high enough to understand the task, was significantly lower than their Spanish mastery. However, recent studies on foreign language effects provide a plausible explanation of why this can be the case. As described in the Introduction [26], different alternative hypotheses could explain why a foreign language would favor a more deliberate and analytic way of thinking [8,11,47]. Potentially, as the non-native language is intrinsically harder to process than the native one, it would demand additional attention resources from the students set in a foreign language context. As compared to their peers in a native language context, they might have been more focused and they might have activated more analytic processes [48], although the difficulty of processing a foreign language and an increased cognitive load is expected to cause the opposite pattern [20,21].

The differences found between the foreign and native language contexts could also be partially explained by the reduced emotional reactivity related to foreign language context [11,49,50]. As Vives and colleagues [26] defended, this seems to occur when the emotions play a causal role in the situation, which was not the case in the recycling part of the task. However, it should be kept in mind that the distracting task (building a tower) did have an emotional component since competitiveness was fostered by promising a prize to the highest tower builder. Therefore, the students in the native language context could have been more emotionally driven and focused on winning (building the highest tower), thus allocating less resources or being more heuristically biased to the disposal instructions, as it did not matter for obtaining the prize. Non-native context learners, being emotionally detached from the situation, would have followed a more systematic process in which they approached all the assignments (task and disposal rules) in a more analytic way.

Nevertheless, the aim of the present study was not to explore the potential origins of the FLE but to find instrumental ways of using language context to improve behavior. Thus, irrespectively of what caused the FLE, the results clearly showed that people who were exposed a single time to a series of recycling rules in their non-native language, complied with them better than the ones who were exposed to them in their native language. Whether this outcome was induced by the highest attentional demands of the non-native context associated with a more analytic approach, or whether it was due to an emotional detachment from the context is a matter of future research.

Interestingly, the rule that our participants had to learn was not relevant for the first (and, to them, the main) task that they were required to do. They believed that there was no explicit evaluation of their performance on the recycling task and that there was no direct benefit of completing it correctly. This somehow mirrors the behaviors that Education for Sustainable Development (ESD) promotes, especially when it comes to recycling. There is normally no individual evaluation of recycling behavior, and those who do it properly do not usually receive a direct benefit or an immediate prize. Some studies have suggested that providing a direct benefit (e.g., paying fees depending on their garbage production) has positive effects on recycling (see [51]). The current study extends this conclusion, showing for the first time in a naturalistic context that an indirect manipulation (e.g., manipulating the language of the instructions) can also improve rule learning, which might lead to a better recycling behavior. It is worth noting, however, that our experiment differed from recycling behaviors in that our participants were not given the option not to recycle, as they had to put the blocks in some container. Indeed, in the current piece of work, we have demonstrated an impact of foreign language on rule learning, which then lead to a better recycling behavior, but this can´t be interpreted as a direct impact of language context in recycling behaviors or prosocial attitudes. The results presented here are promising, as we present the first step of what it could be a fruitful line of research, but further research and validation will be needed.

## 5. Conclusions

In the present study, we have presented novel data on how language context can affect recycling-related rule learning and conforming to these rules. Students who were exposed to a series of rules in their non-native language followed them better than those who received them in their first language. We hypothesized that the non-native language situation was less emotionally biased and posed a more difficult context that involved additional attentional resources and induced more rational and analytic procedures. This research opens very interesting pathways in future research to explore the relationship between a foreign language and rule learning. This work also highlights the importance of a second language as means of communication in a globalized world. If the use of languages other than the mother tongues to make decisions is going to be the rule more than the exception in the near future, future research should aim at identifying the situations and contexts in which the use of those languages can impact social outcomes and modify behaviors. This would be particularly important if the strategic use of a foreign language could encourage and reinforce prosocial attitudes, such as sustainable development.

## Supplementary Materials

The following are available online at https://www.mdpi.com/1660-4601/17/11/3784/s1, Table S1: Number of correctly deposited tokens for each participant in the Spanish and English groups for Days 1 and 2 separated by block colour. Five tokens of each colour had to be placed in the corresponding container.
