# Supplementary material for: Recycling in Babel: The Impact of Foreign Languages in Rule Learning
Source: Int J Environ Res Public Health. 2020 May 27;17(11):3784. doi: 10.3390/ijerph17113784 (PMC7311970; doi:10.3390/ijerph17113784)
Supplement: Supplementary file 1 [file ijerph-17-03784-s001.pdf]

Supplementary Materials

Number of correctly deposited tokens for each participant in the Spanish and English groups for Days 1 and 2 separated by block colour. Five tokens of each colour had to be placed in the corresponding container.

Spanish Context

		**Day 1 Number of Hits**			**Day 2 Number of Hits**				
**Participant**	**Group**	**White**	**Red**	**Green**	**White**	**Red**	**Green**	**Day 1 Total**	**Day 2 Total**
1	A	5	5	5	0	5	0	**15**	**5**
2	A	5	5	5	5	5	5	**15**	**15**
3	A	0	0	5	5	5	5	**5**	**15**
4	A	5	5	5	5	5	5	**15**	**15**
5	A	5	0	0	5	5	5	**5**	**15**
6	A	5	5	5	0	0	5	**15**	**5**
7	A	5	5	5	5	5	5	**15**	**15**
8	A	5	5	5	0	5	0	**15**	**5**
9	A	5	5	5	5	5	5	**15**	**15**
10	A	5	5	5	5	5	5	**15**	**15**
11	A	0	5	0	0	5	0	**5**	**5**
12	A	5	5	5	5	5	5	**15**	**15**
13	A	5	5	5	0	5	0	**15**	**5**
14	A	5	5	5	5	5	5	**15**	**15**
15	A	0	0	0	0	0	0	**0**	**0**
16	A	5	5	5	0	5	0	**15**	**5**
17	A	0	0	0	0	0	0	**0**	**0**
18	A	0	0	5	0	0	0	**5**	**0**
19	A	0	5	0	5	5	5	**5**	**15**
20	A	0	5	0	0	5	0	**5**	**5**
21	A	5	5	5	5	5	5	**15**	**15**

22	A	0	0	0	0	0	0	**0**	**0**
23	A	0	0	0	5	5	5	**0**	**15**
24	A	5	5	5	5	5	5	**15**	**15**
25	C	0	0	0	5	5	5	**0**	**15**
26	C	5	5	5	5	5	5	**15**	**15**
27	C	0	0	0	0	5	0	**0**	**5**
28	C	0	0	0	0	0	0	**0**	**0**
29	C	5	0	0	0	0	5	**5**	**5**
30	C	5	5	5	0	0	5	**15**	**5**
31	C	0	5	0	5	0	0	**5**	**5**
32	C	0	0	5	0	0	5	**5**	**5**
33	C	5	5	5	5	5	5	**15**	**15**
34	C	5	0	0	5	0	0	**5**	**5**
35	C	5	5	5	5	5	5	**15**	**15**
36	C	0	5	0	0	5	0	**5**	**5**
37	C	5	0	0	5	5	5	**5**	**15**
38	C	0	0	5	0	0	0	**5**	**0**
39	C	0	0	5	0	0	5	**5**	**5**
40	C	5	0	0	5	0	0	**5**	**5**
41	C	0	0	0	0	0	0	**0**	**0**
42	C	5	5	5	5	5	5	**15**	**15**
43	C	5	0	0	5	5	5	**5**	**15**
44	C	5	5	5	0	5	0	**15**	**5**
45	C	0	5	0	0	5	0	**5**	**5**
46	C	0	0	0	0	5	0	**0**	**5**
47	C	0	0	0	0	5	0	**0**	**5**

English Context

		Day 1 Number of Hits			Day 2 Number of Hits				
**Participant**	**Group**	**Yellow**	**Blue**	**Black**	**Yellow**	**Blue**	**Black**	**Day 1 Total**	**Day 2 Total**
48	B	5	5	5	5	5	5	**15**	**15**
49	B	5	5	5	5	5	5	**15**	**15**
50	B	5	5	5	5	5	5	**15**	**15**

51	B	5	5	5	0	0	5	**15**	**5**
52	B	0	0	0	0	0	0	**0**	**0**
53	B	5	5	5	5	5	5	**15**	**15**
54	B	5	5	5	5	5	5	**15**	**15**
55	B	5	5	5	5	5	5	**15**	**15**
56	B	5	5	5	5	5	5	**15**	**15**
57	B	0	0	5	0	0	0	**5**	**0**
58	B	0	5	0	5	5	5	**5**	**15**
59	B	5	5	5	5	5	5	**15**	**15**
60	B	5	5	5	5	5	5	**15**	**15**
61	B	5	5	5	5	5	5	**15**	**15**
62	B	0	0	5	0	0	0	**5**	**0**
63	B	5	5	5	5	5	5	**15**	**15**
64	B	5	5	5	5	5	5	**15**	**15**
65	B	5	5	5	5	5	5	**15**	**15**
66	B	5	5	5	5	5	5	**15**	**15**
67	B	0	0	5	0	5	0	**5**	**5**
68	B	0	5	0	0	5	0	**5**	**5**
69	B	5	5	5	0	0	5	**15**	**5**
70	D	5	5	5	5	5	5	**15**	**15**
71	D	5	5	5	0	5	0	**15**	**5**
72	D	5	5	5	0	5	0	**15**	**5**
73	D	5	5	5	5	5	5	**15**	**15**
74	D	5	5	5	5	5	5	**15**	**15**
75	D	5	5	5	0	0	5	**15**	**5**
76	D	5	5	5	5	5	5	**15**	**15**
77	D	5	5	5	0	5	0	**15**	**5**
78	D	5	5	5	0	0	5	**15**	**5**
79	D	0	0	0	0	0	0	**0**	**0**
80	D	5	5	5	0	0	5	**15**	**5**
81	D	0	0	0	5	5	5	**0**	**15**
82	D	5	5	5	5	5	5	**15**	**15**
83	D	0	0	5	5	5	5	**5**	**15**

84	D	5	5	5	5	5	5	**15**	**15**
85	D	5	5	5	5	5	5	**15**	**15**
86	D	5	5	5	0	0	5	**15**	**5**
87	D	5	5	5	0	5	0	**15**	**5**
88	D	5	5	5	5	5	5	**15**	**15**
89	D	5	5	5	5	5	5	**15**	**15**
90	D	0	0	5	5	5	5	**5**	**15**
